# Supplementary material for: Physical and mental effects of different radical prostatectomy techniques on urologic surgeons
Source: PeerJ. 2025 Aug 20;13:e19908. doi: 10.7717/peerj.19908 (PMC12374686; doi:10.7717/peerj.19908)
Supplement: Supplemental Information 1 [file peerj-13-19908-s001.docx]

**Supplemental Table 1.**  Correlation between complaints and sport/exercise activities.

|  |  | Walking | Running | Bicycling | Swimming | Football | Basketball | Volleyball | Tennis | Golf | Weight  Lifting | Boxing | Meditation | Yoga | Pilates | Total  Exercises |
| --- | --- | --- | --- | --- | --- | --- | --- | --- | --- | --- | --- | --- | --- | --- | --- | --- |
| Pyhsical Exhaustion | ***C.C (r)*** | 0,006 | 0,077 | -0,001 | -0,003 | 0,195 | 0,006 | 0,137 | 0,004 | -0,013 | 0,051 | 0,114 | 0,011 | 0,007 | -0,120 | 0,059 |
|  | ***p*** | 0,930 | 0,258 | 0,984 | 0,966 | **0,004** | 0,926 | **0,042** | 0,954 | 0,851 | 0,452 | 0,092 | 0,870 | 0,921 | 0,076 | 0,386 |
| Forehead Pain | ***C.C (r)*** | 0,055 | 0,054 | 0,036 | -0,094 | 0,009 | 0,053 | 0,076 | 0,064 | -0,052 | 0,067 | -0,016 | 0,015 | -0,021 | 0,007 | 0,052 |
|  | ***p*** | 0,421 | 0,423 | 0,594 | 0,167 | 0,889 | 0,435 | 0,264 | 0,343 | 0,443 | 0,322 | 0,808 | 0,829 | 0,753 | 0,921 | 0,446 |
| Eye Strain | ***C.C (r)*** | 0,098 | 0,100 | -0,038 | -0,012 | 0,025 | -0,014 | 0,089 | 0,082 | -0,062 | 0,188 | -0,030 | 0,111 | 0,065 | -0,035 | 0,125 |
|  | ***p*** | 0,146 | 0,139 | 0,577 | 0,858 | 0,710 | 0,840 | 0,190 | 0,227 | 0,357 | **0,005** | 0,659 | 0,100 | 0,337 | 0,610 | 0,065 |
| Neck Pain | ***C.C (r)*** | 0,012 | 0,029 | 0,008 | 0,038 | -0,021 | 0,098 | 0,032 | 0,031 | -0,056 | 0,125 | 0,012 | 0,039 | 0,079 | 0,004 | 0,075 |
|  | ***p*** | 0,861 | 0,672 | 0,900 | 0,577 | 0,762 | 0,147 | 0,638 | 0,652 | 0,405 | 0,064 | 0,862 | 0,561 | 0,244 | 0,950 | 0,271 |
| Back Pain | ***C.C (r)*** | 0,087 | -0,020 | 0,065 | -0,077 | 0,015 | -0,047 | -0,022 | 0,029 | -0,071 | 0,133 | -0,001 | -0,019 | -0,012 | 0,010 | 0,052 |
|  | ***p*** | 0,200 | 0,765 | 0,337 | 0,254 | 0,820 | 0,484 | 0,741 | 0,666 | 0,294 | **0,050** | 0,989 | 0,777 | 0,858 | 0,884 | 0,440 |
| Shoulder stiffness | ***C.C (r)*** | 0,086 | 0,124 | 0,033 | 0,009 | -0,031 | 0,054 | 0,023 | 0,010 | -0,068 | 0,183 | -0,071 | -0,004 | -0,027 | 0,076 | 0,122 |
|  | ***p*** | 0,205 | 0,067 | 0,632 | 0,893 | 0,651 | 0,428 | 0,738 | 0,879 | 0,315 | **0,007** | 0,295 | 0,955 | 0,691 | 0,263 | 0,070 |
| Chest Pain | ***C.C (r)*** | 0,218 | 0,112 | 0,016 | -0,085 | 0,099 | 0,122 | -0,035 | 0,050 | -0,028 | 0,114 | -0,057 | -0,010 | -0,039 | 0,014 | 0,151 |
|  | ***p*** | **0,001** | 0,097 | 0,813 | 0,208 | 0,143 | 0,072 | 0,604 | 0,458 | 0,682 | 0,091 | 0,402 | 0,886 | 0,570 | 0,831 | **0,025** |
| Arm Pain | ***C.C (r)*** | 0,067 | 0,072 | 0,034 | 0,024 | 0,037 | 0,137 | 0,002 | 0,084 | -0,025 | 0,089 | -0,073 | -0,061 | -0,056 | 0,023 | 0,090 |
|  | ***p*** | 0,325 | 0,284 | 0,616 | 0,723 | 0,590 | **0,042** | 0,980 | 0,217 | 0,713 | 0,188 | 0,278 | 0,371 | 0,407 | 0,733 | 0,184 |
| Forearm Pain | ***C.C (r)*** | 0,130 | 0,094 | -0,003 | -0,064 | 0,056 | 0,180 | 0,008 | 0,110 | -0,018 | 0,173 | -0,059 | -0,049 | -0,057 | -0,025 | 0,117 |
|  | ***p*** | 0,053 | 0,165 | 0,960 | 0,343 | 0,405 | **0,008** | 0,901 | 0,104 | 0,793 | **0,010** | 0,383 | 0,469 | 0,402 | 0,710 | 0,084 |
| Elbow Stiffness | ***C.C (r)*** | 0,138 | 0,214 | -0,015 | 0,022 | 0,050 | 0,083 | 0,159 | 0,077 | 0,058 | 0,301 | 0,063 | 0,140 | 0,118 | -0,002 | 0,257 |
|  | ***p*** | **0,040** | **0,001** | 0,823 | 0,744 | 0,457 | 0,219 | **0,019** | 0,256 | 0,392 | **0,000** | 0,354 | 0,038 | 0,080 | 0,975 | **<0,001** |
| Hand Pain | ***C.C (r)*** | 0,115 | 0,102 | -0,014 | -0,008 | 0,053 | 0,274 | 0,102 | 0,125 | 0,049 | 0,128 | -0,015 | -0,029 | 0,007 | -0,018 | 0,147 |
|  | ***p*** | 0,088 | 0,132 | 0,838 | 0,903 | 0,434 | **<0,001** | 0,132 | 0,064 | 0,467 | 0,058 | 0,822 | 0,673 | 0,921 | 0,786 | **0,030** |
| Wrist Stiffness | ***C.C (r)*** | 0,051 | 0,043 | 0,015 | -0,086 | -0,041 | 0,226 | -0,039 | 0,019 | -0,059 | 0,148 | -0,093 | -0,028 | -0,048 | 0,012 | 0,045 |
|  | ***p*** | 0,448 | 0,527 | 0,825 | 0,203 | 0,549 | **0,001** | 0,568 | 0,781 | 0,382 | **0,028** | 0,171 | 0,678 | 0,474 | 0,857 | 0,506 |
| Finger Numbness | ***C.C (r)*** | 0,067 | 0,200 | -0,038 | 0,014 | 0,010 | 0,178 | 0,008 | 0,065 | -0,020 | 0,174 | -0,068 | 0,032 | 0,029 | 0,032 | 0,143 |
|  | ***p*** | 0,320 | **0,003** | 0,574 | 0,841 | 0,878 | **0,008** | 0,909 | 0,336 | 0,769 | **0,010** | 0,315 | 0,641 | 0,672 | 0,639 | **0,033** |
| Leg Pain | ***C.C (r)*** | 0,103 | 0,112 | -0,044 | 0,024 | 0,088 | 0,131 | 0,084 | 0,123 | 0,059 | 0,174 | 0,156 | -0,027 | 0,157 | -0,051 | 0,172 |
|  | ***p*** | 0,126 | 0,097 | 0,517 | 0,724 | 0,193 | 0,052 | 0,216 | 0,068 | 0,388 | **0,010** | **0,021** | 0,686 | **0,020** | 0,455 | **0,011** |
| Total Complaints | ***C.C (r)*** | 0,129 | 0,135 | 0,009 | -0,030 | 0,033 | 0,148 | 0,047 | 0,093 | -0,044 | 0,233 | -0,025 | 0,012 | 0,028 | 0,008 | 0,170 |
|  | ***p*** | 0,056 | **0,045** | 0,895 | 0,663 | 0,629 | **0,028** | 0,491 | 0,169 | 0,517 | **0,001** | 0,717 | 0,855 | 0,678 | 0,906 | **0,012** |

*C.C, correlation coefficient
