# Supplementary material for: Physical and mental effects of different radical prostatectomy techniques on urologic surgeons
Source: PeerJ. 2025 Aug 20;13:e19908. doi: 10.7717/peerj.19908 (PMC12374686; doi:10.7717/peerj.19908)
Supplement: Supplemental Information 2 [file peerj-13-19908-s002.pdf]

ERGONOMIC EVALUATION OF RADICAL PROSTATECTOMY TECHNIQUES

A total of 10 questions are in the survey.
The estimated time to complete the survey is approximately 5 minutes.

* 1. How many radical prostatectomy surgeries do you perform a year? (Please check all that apply)

Open	
Laparoscopic	
Robotic	

* 2. Before the radical prostatectomy, Do your patients and/or their family (relatives) cause stress on you?

- Always
- Usually
- Sometimes
- Rarely
- Never

* 3. How often do expectations of your employer about radical prostatectomy cause stress on you?

- Always
- Usually
- Sometimes
- Rarely
- Never

* 4. During a radical prostatectomy, which of the following(s) cause(s) stress on you? (Please check all that apply)

- None
- Patient with morbid obesity
- Grade or stage of the disease
- Prior history of abdominal operation
- Other (please specify)
- Frequent change of the surgical team
- Complications during or after the surgery
- Lack of standard postoperative care
- Postoperative follow-up

* 5. How much does/do the preferred technique(s) exhaust you physically? (Please check all that apply)

	Severity score (0 - 10)
Open	
Laparoscopic	
Robotic	

6. Which of these complaints do you experience during or after performing radical prostatectomy with the preferred technique(s)? (Please specify the severity of your complaints between "1 and 10". (**If you do not experience any complaint, skip this question**. Please check all that apply)

	Open	Lap	Robotic
Forehead pain			
Eye strain			
Neck pain			
Back pain			
Shoulder stiffness			
Chest pain			
Arm pain			
Forearm pain			
Elbow stiffness			
Hand pain			
Wrist stiffness			
Finger numbness			
Leg pain			

Other (please specify the preferred technique, your complaints, and the severity between 1 and 10)

7. Have you received any professional support for the complaints that you have experienced? (**If you do not experience any complaint, skip this question**. Please check all that apply)

- ☐ No
- ☐ Lifestyle modifications
- ☐ Physical therapy modalities (Massage, TENS, dry needling, hot-cold compress, stretching)
- ☐ Medical treatment(s)
- ☐ Surgery
- ☐ Other (please specify)

8. Do/Does your complaint(s) affect your choice of radical prostatectomy technique? **(If you do not experience any complaint, skip this question.)**

- ○ Always
- ○ Usually
- ○ Sometimes
- ○ Rarely
- ○ Never
- ○ I can perform only one type of technique.

9. Please only choose the exercise and/or physical activities which you **regularly** do (**If none is regular, skip this question**. Please check all that apply)

	times/week
Walking	
Running	
Bicycling	
Swimming	
Football	
Basketball	
Volleyball	
Tennis	
Golf	
Weightlifting	
Boxing	
Meditation	
Yoga	
Pilates	

Other (please specify your sports activity or exercise type with its frequency within a week)

* 10. Please specify your age, gender, weight, height, and country

Age (year):

Gender:

Weight (kg):

Height (cm):

Country:
