# Supplementary material for: Physical and mental effects of different radical prostatectomy techniques on urologic surgeons
Source: PeerJ. 2025 Aug 20;13:e19908. doi: 10.7717/peerj.19908 (PMC12374686; doi:10.7717/peerj.19908)
Supplement: Supplemental Information 3 [file peerj-13-19908-s003.pdf]

**Daha önce bu anketin ingilizce formatlı olanına katıldıysanız lütfen bu anketi doldurmayınız.**

**Anket toplamda 10 sorudan oluşmaktadır.**

**Anketin tahmini tamamlanma süresi yaklaşık 5 dakika olarak öngörülmüştür.**

* 1. Bir yılda yaklaşık olarak kaç radikal prostatektomi ameliyatı yapıyorsunuz? (Birden fazla seçebilirsiniz)

| | |
|---|---|
| Açık | |
| Laparoskopik | |
| Robotik | |

* 2. Radikal prostatektomi öncesi hasta ve/veya hasta yakınlarının beklentileri sizde ne sıklıkta strese neden olmaktadır?

- Her zaman
- Genellikle
- Bazen
- Nadiren
- Hiçbir zaman

* 3. İşvereninizin radikal prostatektomi konusundaki beklentileri sizde ne sıklıkta strese neden olmaktadır?

- Her zaman
- Genellikle
- Bazen
- Nadiren
- Hiçbir zaman

* 4. Radikal prostatektomi esnasında aşağıdakilerden hangileri sizde strese neden olmaktadır? (Birden fazla seçebilirsiniz)

- Hiçbiri
- Hastanın morbid obez olması
- Hastalığın derecesi yada evresi
- Önceden geçirilmiş batın cerrahisi
- Diğer (lütfen belirtiniz)
- Operasyon esnasında cerrahi ekibin sıkça değişmesi
- Operasyon esnasında yada sonrasındaki komplikasyonlar
- Postoperatif bakım ünitesi (derlenme) yetersizliği
- Operasyon sonrası takip

* 5. Tercih ettiğiniz radikal prostatektomi teknik(ler)i sizi fiziksel olarak ne derece yormaktadır? (Birden fazla seçebilirsiniz)

| | Skor (0 - 10 ) |
|---|---|
| Açık | |
| Laparoskopik | |
| Robotik | |

6. Aşağıdaki şikayetlerden hangileri tercih ettiğiniz radikal prostatektomi teknik(ler) i sırasında sizde olmaktadır? (Lütfen şikayetlerinizin şiddetini "1-10"arasında belirtiniz. **Eğer herhangi bir şikayetiniz yoksa bu soruyu boş bırakınız**. Birden fazla seçebilirsiniz)

| | Açık | Lap | Robotik |
|---|---|---|---|
| Baş ağrısı | | | |
| Göz yorgunluğu | | | |
| Boyun ağrısı | | | |
| Bel ağrısı | | | |
| Omuz sertliği | | | |
| Göğüs ağrısı | | | |
| Kol ağrısı | | | |
| Ön kol ağrısı | | | |
| Dirsek sertliği | | | |
| El ağrısı | | | |
| El bileği sertliği | | | |
| Parmak uyuşması | | | |
| Bacak ağrısı | | | |

Diğer ( Lütfen tercih ettiğiniz teknik(ler), şikayetleriniz ve şikayet şiddetini 1-10 arasında belirtiniz)

7. Şikayetleriniz için profesyonel bir destek aldınız mı? (**Eğer herhangi bir şikayetiniz yoksa bu soruyu boş bırakınız**. Birden fazla seçebilirsiniz)

- [ ] Hayır
- [ ] Yaşam tarzı modifikasyonları
- [ ] Fizik tedavi modaliteleri ( Masaj, TENS, kuru iğneleme, sıcak-soğuk kompres, germe ...vb)
- [ ] Medikal tedavi(ler)
- [ ] Cerrahi
- [ ] Diğer (Lütfen belirtiniz)

8. Şikayetleriniz radikal prostatektomi teknik seçiminize etki ediyor mu? (**Eğer herhangi bir şikayetiniz yoksa bu soruyu boş bırakınız**)

- ○ Her zaman
- ○ Genellikle
- ○ Bazen
- ○ Nadiren
- ○ Asla
- ○ Sadece bir teknikle radikal prostatektomi yapabiliyorum

9. Lütfen düzenli olarak yaptığınız egzersizlerin ve/veya sporların sıklığını belirtiniz. (**Eğer düzenli olarak egzersiz yada spor yapmıyorsanız bu soruyu boş bırakınız**. Birden fazla seçebilirsiniz)

| | kez /hafta |
|---|---|
| Yürüme | |
| Koşma | |
| Bisiklet sürme | |
| Yüzme | |
| Futbol | |
| Basketbol | |
| Voleybol | |
| Tenis | |
| Golf | |
| Ağırlık kaldırma | |
| Boks | |
| Meditasyon | |
| Yoga | |
| Pilates | |

Diğer ( Lütfen düzenli olarak , bir hafta içinde yaptığınız egzersizleri ve/veya sporları sıklığı ile birlikte belirtiniz )

* 10. Lütfen yaşınızı, cinsiyetinizi, ağırlığınızı, boyunuzu ve yaşadığınız ülkeyi belirtiniz.

Yaş (yıl) :

Cinsiyet :

Ağırlık (kg) :

Boy (cm) :

Ülke :
